# Quercetin Attenuates Brain Oxidative Alterations Induced by Iron Oxide Nanoparticles in Rats

**DOI:** 10.3390/ijms22083829

**Published:** 2021-04-07

**Authors:** Mohamed F. Dora, Nabil M. Taha, Mohamed A. Lebda, Aml E. Hashem, Mohamed S. Elfeky, Yasser S. El-Sayed, Soad Al Jaouni, Ali H. El-Far

**Affiliations:** 1 Department of Biochemistry, Faculty of Veterinary Medicine, Alexandria University, Alexandria 21526, Egypt; MohamedDoravet@alexu.edu.eg (M.F.D.); Prof_nabil2006@yahoo.com (N.M.T.); amlhashem@alexu.edu.eg (A.E.H.); elfeky@alexu.edu.eg (M.S.E.); 2 Department of Forensic Medicine and Toxicology, Faculty of Veterinary Medicine, Damanhur University, Damanhour 22511, Egypt; elsayed-ys@vetmed.dmu.edu.eg; 3 Department of Hematology/Pediatric Oncology, Yousef Abdulatif Jameel Scientific Chair of Prophetic Medicine Application, Faculty of Medicine, King Abdulaziz University, Jeddah 21589, Saudi Arabia; saljaouni@kau.edu.sa; 4 Department of Biochemistry, Faculty of Veterinary Medicine, Damanhour University, Damanhour 22511, Egypt; ali.elfar@damanhour.edu.eg

**Keywords:** iron oxide nanoparticles, oxidative stress, quercetin, anti-oxidant

## Abstract

Iron oxide nanoparticle (IONP) therapy has diverse health benefits but high doses or prolonged therapy might induce oxidative cellular injuries especially in the brain. Therefore, we conducted the current study to investigate the protective role of quercetin supplementation against the oxidative alterations induced in the brains of rats due to IONPs. Forty adult male albino rats were allocated into equal five groups; the control received a normal basal diet, the IONP group was intraperitoneally injected with IONPs of 50 mg/kg body weight (B.W.) and quercetin-treated groups had IONPs + Q25, IONPs + Q50 and IONPs + Q100 that were orally supplanted with quercetin by doses of 25, 50 and 100 mg quercetin/kg B.W. daily, respectively, administrated with the same dose of IONPs for 30 days. IONPs induced significant increases in malondialdehyde (MDA) and significantly decreased reduced glutathione (GSH) and oxidized glutathione (GSSG). Consequently, IONPs significantly induced severe brain tissue injuries due to the iron deposition leading to oxidative alterations with significant increases in brain creatine phosphokinase (CPK) and acetylcholinesterase (AChE). Furthermore, IONPs induced significant reductions in brain epinephrine, serotonin and melatonin with the downregulation of peroxisome proliferator-activated receptor gamma coactivator 1-alpha (*PGC-1α*) and mitochondrial transcription factor A (*mtTFA*) mRNA expressions. IONPs induced apoptosis in the brain monitored by increases in caspase 3 and decreases in B-cell lymphoma 2 (Bcl2) expression levels. Quercetin supplementation notably defeated brain oxidative damages and in a dose-dependent manner. Therefore, quercetin supplementation during IONPs is highly recommended to gain the benefits of IONPs with fewer health hazards.

## 1. Introduction

Iron oxide nanoparticles (IONPs) are used for drug targeting, gene delivery, cell labeling, a contrast agent in magnetic resonance imaging and hyperthermia therapy [1]. It is also used as a food additive in iron-fortified drinks and cereals for human consumption [2] besides numerous industrial uses in wastewater treatment, gas sensing and semiconductors sorbents lubrications, pigments and coatings [3]. However, the diverse usage of IONPs induces iron overload and the subsequent initiation of oxidative stress in various organs [4]. Ingestion, inhalation and dermal penetration are the main routes for the entry of IONPs [5]. In the bloodstream, IONPs are bound to plasma proteins and distributed into different body organs including the liver, spleen, kidney, lung and heart and penetrate the blood-brain barrier (BBB), inducing oxidative injuries [6,7]. Irons released from IONPs causing reactive oxygen species (ROS) generation lead to serious oxidative alterations in lipids, proteins and DNA [8,9].

Quercetin (3, 3′, 4′, 5, 7-pentahydroxyflavone) is a flavonoid present in fruits (apples, berries, cherries and red grapes) and vegetables (onions and broccoli) in addition to many seeds, buckwheat, nuts, flowers, barks, green tea and olive oil [10]. Quercetin has plentiful valuable effects comprising anti-inflammatory, anti-oxidant, anti-mutagenic, anti-ischemic, anti-viral and anti-aging effects [11,12,13,14,15,16,17]. Quercetin is lipophilic and can penetrate the blood-brain barrier (BBB) [18]. Furthermore, quercetin exhibits a potential anti-oxidant role by the enhancement of reduced glutathione (GSH) and the upregulation of copper-zinc superoxide dismutase (SOD1) [17]. Quercetin can chelate iron, inhibiting Fenton’s reaction and inhibiting ROS generation [19]. Consequently, quercetin is a potential therapeutic for multiple neurodegenerative diseases and neuronal injuries [20]. The anti-oxidant potential of quercetin encouraged us to conduct the present study to investigate the protective potential of quercetin against the oxidative alterations induced by IONPs on brain tissues.

## 2. Results

### 2.1. Size and Charge of IONPs

**Figure 1:**
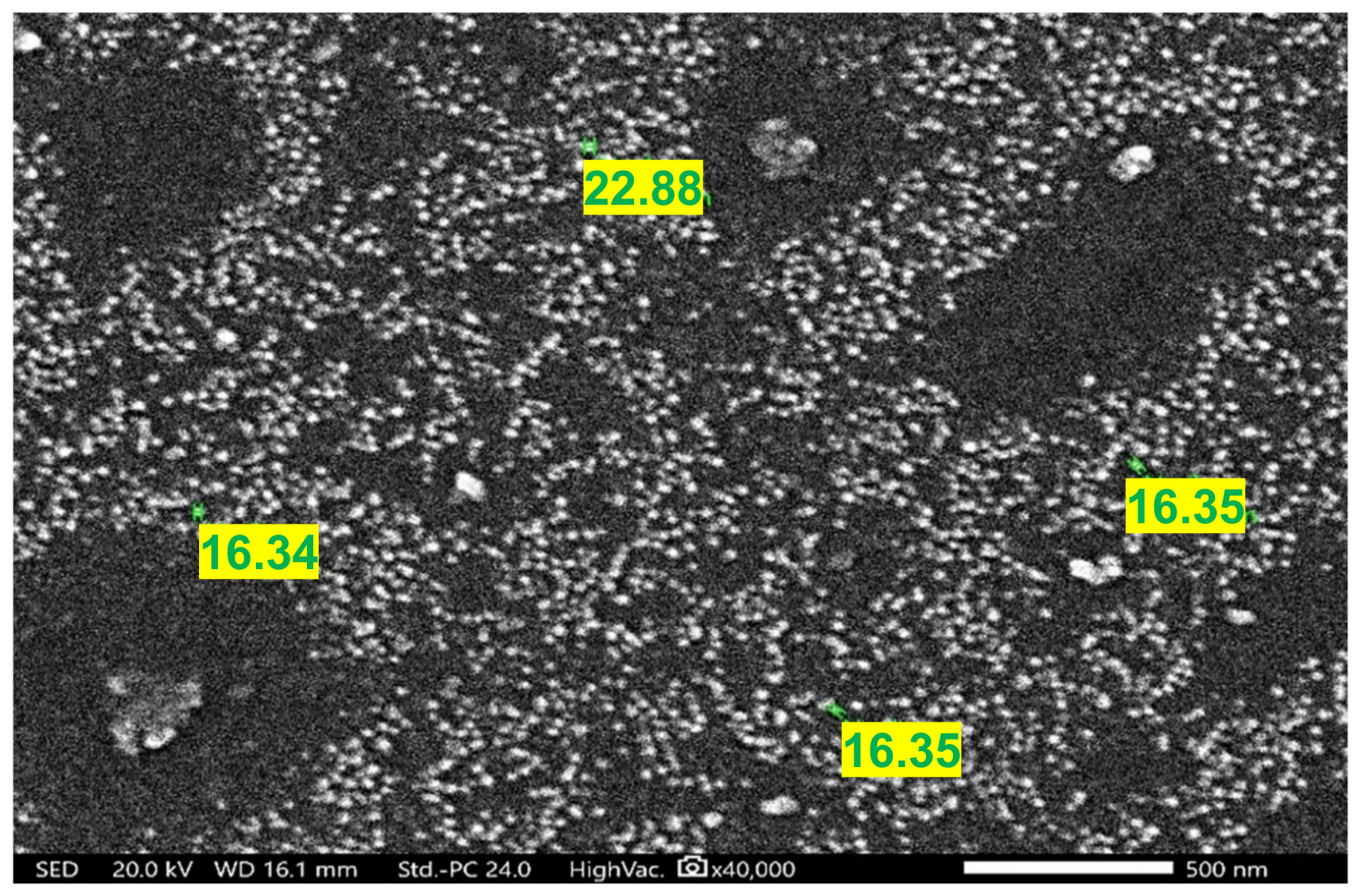
SEM image of iron oxide nanoparticles (IONPs). IONPs were spherical in shape with an average size of 16.34–22.88 nm. The scale bar represents 500 nm.

**Figure 2:**
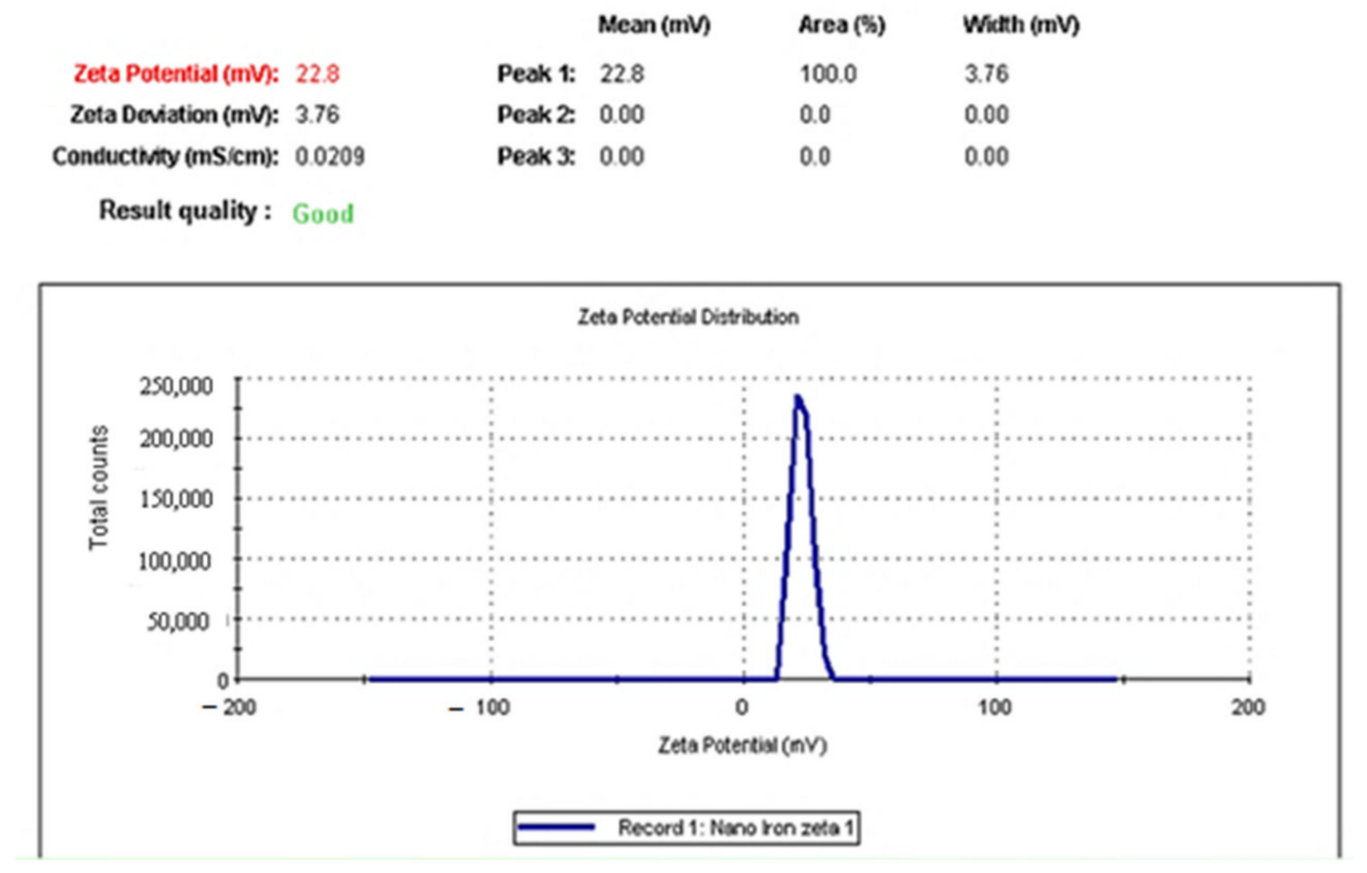
IONPs had a positive zeta potential value of +22.8 mV.

A SEM image of IONPs, as shown in Figure 1, revealed a spherical shape with an average size 16.34–22.88 nm while possessing a positive zeta potential value +22.8 mV (Figure 2).

### 2.2. Brain Oxidative Stress and Anti-Oxidant Status

**Figure 3:**
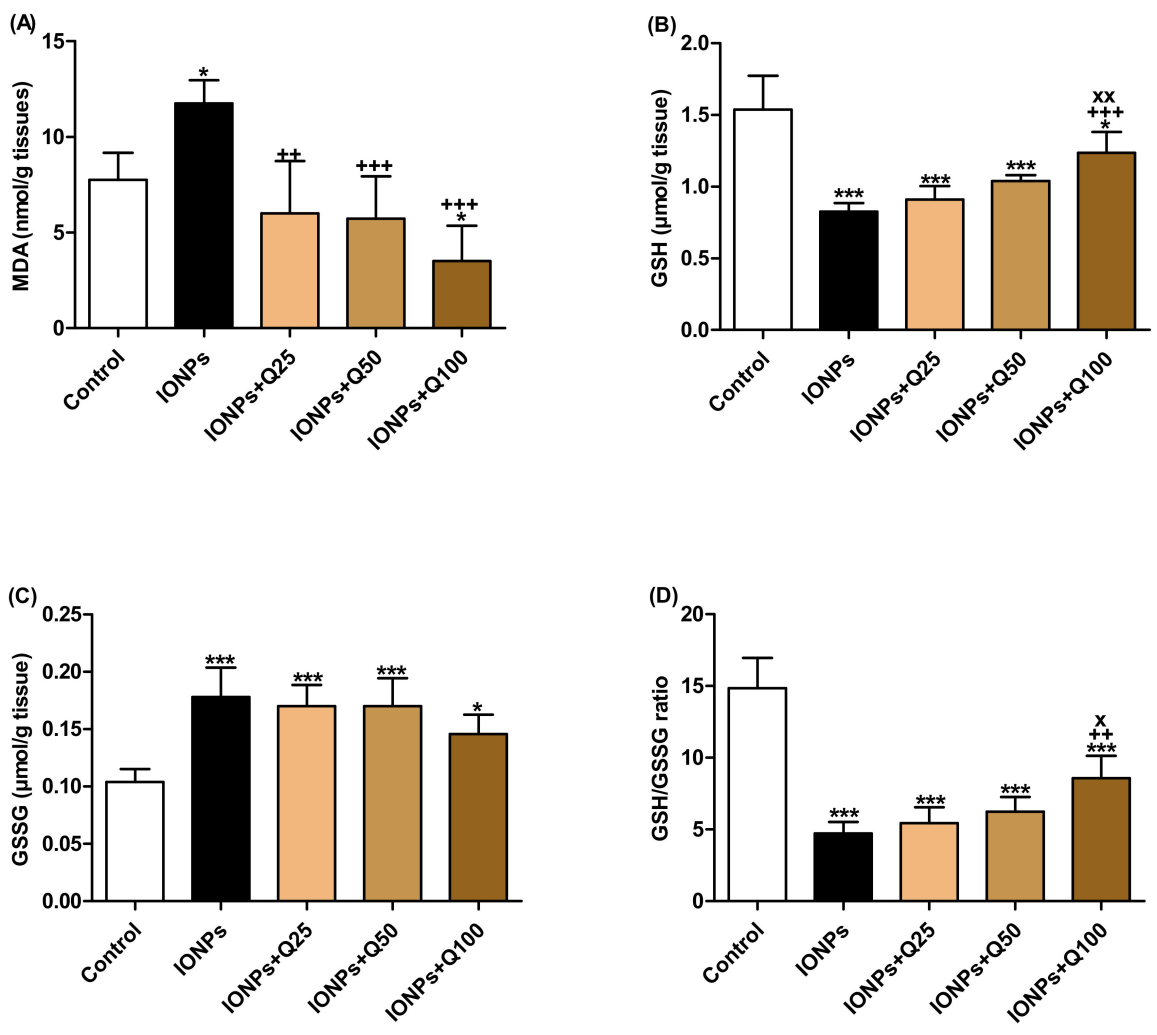
The oxidative stress and anti-oxidant status of brain tissues. (**A**) Malondialdehyde (MDA) (nmol/g tissues). (**B**) Glutathione (GSH) (µmol/g tissue). (**C**) Oxidized glutathione (GSSG) (µmol/g tissue). (**D**) GSH/GSSG ratio. Data were analyzed with a one-way ANOVA followed by Tukey’s multiple comparison test. * *p* < 0.05 and *** *p* < 0.001 vs. the control. ^++^ *p* < 0.01 and ^+++^ *p* < 0.001 vs. IONPs. ^x^ *p* < 0.05 and ^xx^ *p* < 0.01 vs. IONPs + Q25. Error bars represent mean ± SD. *n* = 5. White color column refers to control. Black color column refers to IONPs. Colored column with different extents refers to different concentrations of quercetin supplementations to IONPs-treated groups.

The levels of the lipid peroxidation product malondialdehyde (MDA) were significantly increased in the IONPs group (*p* < 0.05) while they significantly decreased in IONPs + Q100 (*p* < 0.05) compared with the control (Figure 3A). MDA levels in IONPs + Q25 (*p* < 0.01), IONPs + Q50 (*p* < 0.01) and IONPs + Q100 (*p* < 0.01) were significantly decreased compared with IONPs.

The GSH levels in brain homogenate were significantly decreased in IONPs (*p* < 0.001), IONPs + Q25 (*p* < 0.001), IONPs + Q50 (*p* < 0.001) and IONPs + Q100 (*p* < 0.05) compared with the control group. In IONPs + Q100, the GSH levels were significantly increased than IONPs (*p* < 0.001) and IONPs + Q25 (*p* < 0.01), as represented in Figure 3B.

The oxidized glutathione (GSSG) levels were significantly increased in IONPs (*p* < 0.001), IONPs + Q25 (*p* < 0.001), IONPs + Q50 (*p* < 0.001) and IONPs + Q100 (*p* < 0.05) compared with the control group (Figure 3C).

Based on the data of GSH and GSSG, the GSH/GSSG ratio values were significantly decreased in IONPs (*p* < 0.001), IONPs + Q25, IONPs + Q50 and IONPs + Q100 compared with the control group (Figure 3D). In IONPs + Q100, GSH/GSSG ratio values were significantly increased in comparison with IONPs (*p* < 0.01) and IONPs + Q25 (*p* < 0.05).

### 2.3. Brain Creatine Phosphokinase (CPK) and Acetylcholinesterase (AChE) Activities

**Figure 4:**
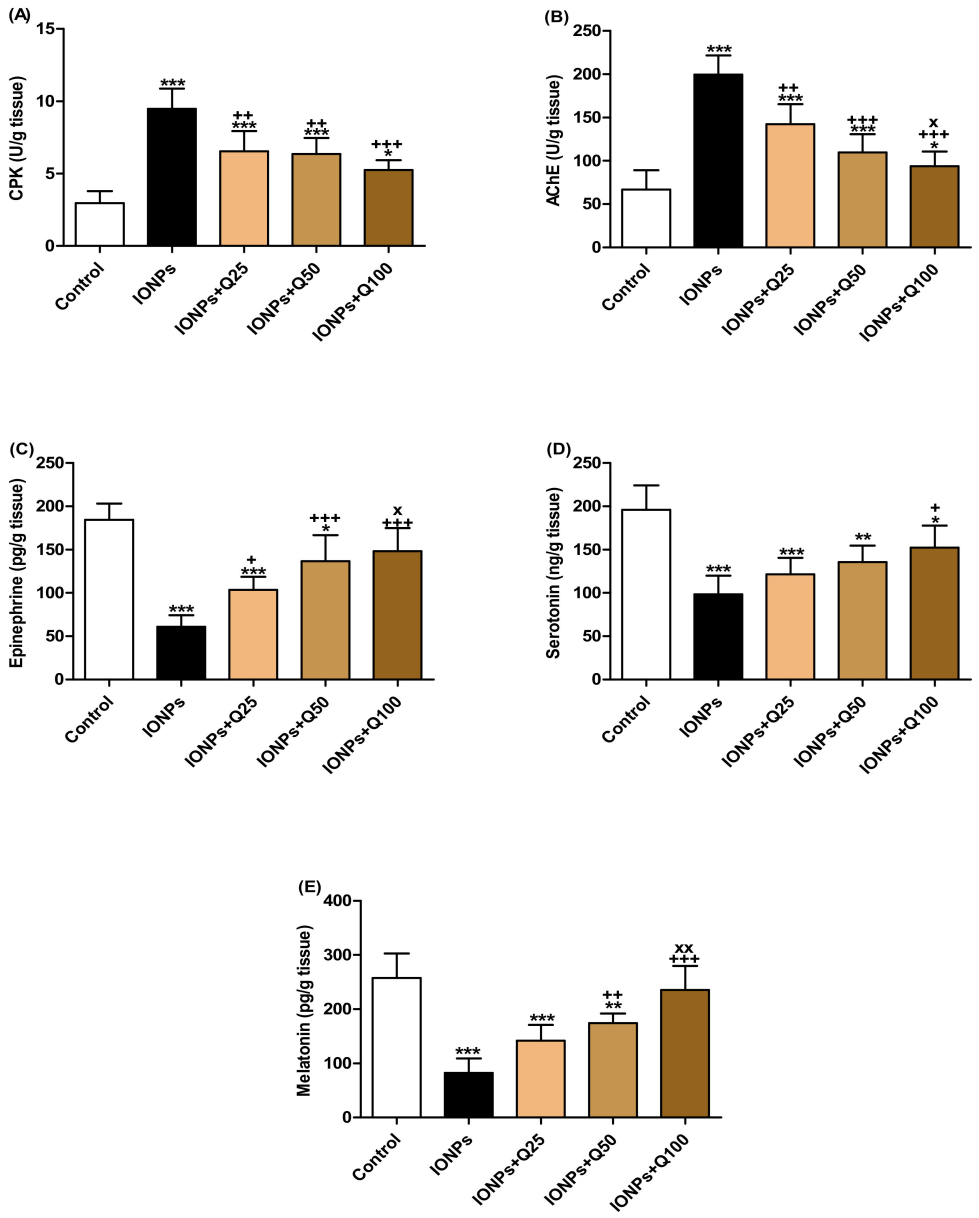
Biochemical assessments of brain tissue. (**A**) Creatine phosphokinase (CPK) (U/g tissue). (**B**) Acetylcholinesterase (AChE) (U/g tissue). (**C**) Epinephrine (pg/g tissue). (**D**) Serotonin (ng/g tissue). (**E**) Melatonin (pg/g tissue). Data were analyzed with a one-way ANOVA followed by Tukey’s multiple comparison test. * *p* < 0.05, ** *p* < 0.01 and *** *p* < 0.001 vs. the control. ^+^ *p* < 0.05, ^++^ *p* < 0.01 and ^+++^ *p* < 0.001 vs. IONPs. ^x^ *p* < 0.05 and ^xx^ *p* < 0.01 vs. IONPs + Q25. Error bars represent mean ± SD. *n* = 5. White color column refers to control. Black color column refers to IONPs. Colored column with different extents refers to different concentrations of quercetin supplementations to IONPs-treated groups.

CPK activities were significantly increased in IONPs (*p* < 0.001), IONPs + Q25 (*p* < 0.001), IONPs + Q50 (*p* < 0.001) and IONPs + Q100 (*p* < 0.05) compared with the control group. In comparison with the IONP groups, CPK activities were significantly decreased in IONPs + Q25 (*p* < 0.01), IONPs + Q50 (*p* < 0.01) and IONPs + Q100 (*p* < 0.001) (Figure 4A).

In Figure 4B, AChE activities were significantly increased in IONPs (*p* < 0.001), IONPs + Q25 (*p* < 0.001), IONPs + Q50 (*p* < 0.001) and IONPs + Q100 (*p* < 0.05) compared with the control group. They significantly decreased in IONPs + Q25 (*p* < 0.01), IONPs + Q50 (*p* < 0.001) and IONPs + Q100 (*p* < 0.001) than IONPs. In addition, AChE activities were significantly decreased (*p* < 0.001) in IONPs + Q100 compared with IONPs + Q25.

### 2.4. Brain Epinephrine, Serotonin and Melatonin Hormones

Epinephrine levels in brain homogenates were significantly decreased in IONPs (*p* < 0.001), IONPs + Q25 (*p* < 0.001) and IONPs + Q50 (*p* < 0.05) compared with the control group (Figure 4C). In the IONPs + Q25 (*p* < 0.05), IONPs + Q50 (*p* < 0.001) and IONPs + Q100 (*p* < 0.001) groups, the epinephrine levels were significantly increased compared with IONPs. Furthermore, the levels were significantly increased in IONPs + Q100 (*p* < 0.05) compared with IONPs + Q25.

As can be seen in Figure 4D, serotonin levels were significantly decreased in IONPs (*p* < 0.001), IONPs + Q25 (*p* < 0.001), IONPs + Q50 (*p* < 0.01) and IONPs + Q100 (*p* < 0.05) compared with the control group. In IONPs + Q100, the levels significantly increased (*p* < 0.05) than IONPs + Q25.

Melatonin levels in brain homogenates were significantly decreased in IONPs (*p* < 0.001), IONPs + Q25 (*p* < 0.001) and IONPs + Q50 (*p* < 0.01) compared with the control group (Figure 4E). In comparison with IONPs, the levels in the IONPs + Q50 (*p* < 0.01) and IONPs + Q100 (*p* < 0.001) groups were significantly increased. It significantly increased (*p* < 0.001) in IONPs + Q100 compared with IONPs + Q25.

### 2.5. Brain PGC-1α and mtTFA mRNA Expression

**Figure 5:**
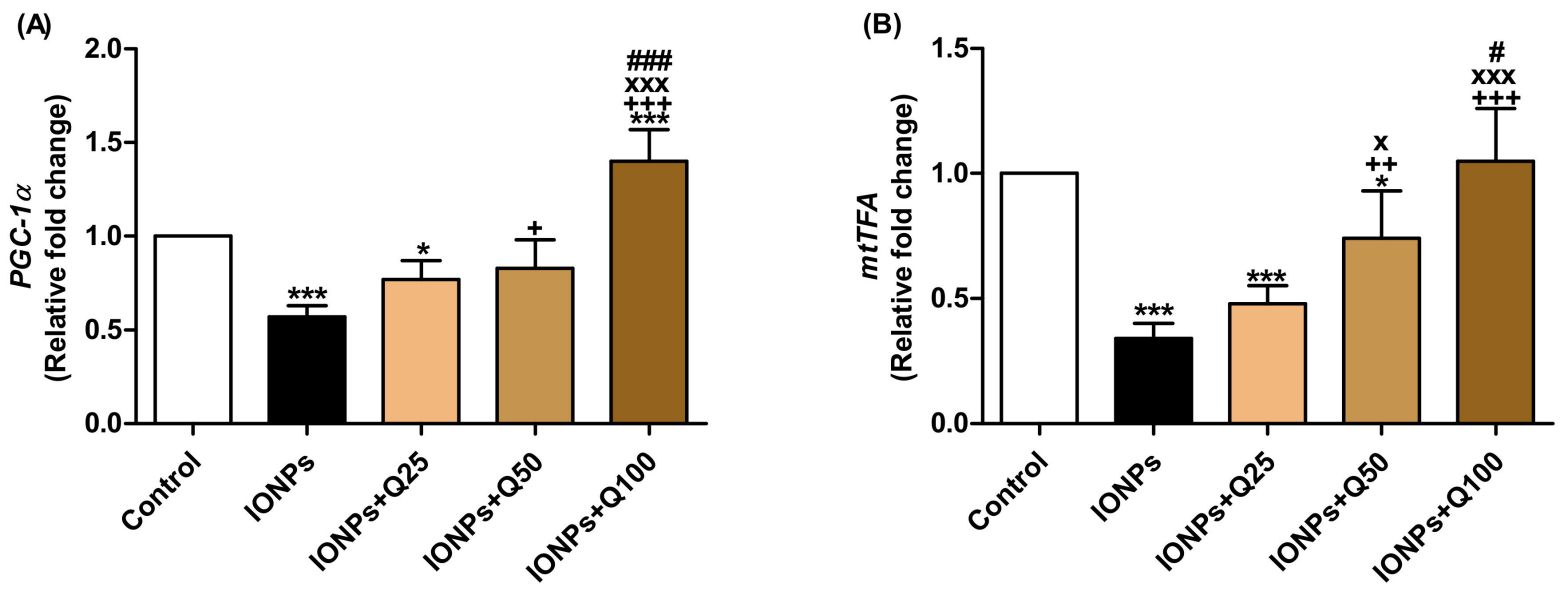
mRNA relative fold change expression of brain tissue. (**A**) *PGC-1α*. (**B**) *mtTFA*. Data were analyzed with a one-way ANOVA followed by Tukey’s multiple comparison test. * *p* < 0.05 and *** *p* < 0.001 vs. the control. ^++^ *p* < 0.01 and ^+++^ *p* < 0.001 vs. IONPs. ^x^ *p* < 0.05 and ^xxx^ *p* < 0.001 vs. IONPs + Q25. ^#^ *p* < 0.05 and ^###^ *p* < 0.001 vs. IONPs + Q25. Error bars represent mean ± SD. *n* = 5. White color column refers to control. Black color column refers to IONPs. Colored column with different extents refers to different concentrations of quercetin supplementations to IONPs-treated groups.

Peroxisome proliferator-activated receptor gamma coactivator 1-alpha (*PGC-1α*) mRNA expression fold changes were significantly decreased in IONPs (*p* < 0.001) and IONPs + Q25 (*p* < 0.05) and significantly increased in IONPs + Q100 (*p* < 0.001) compared with the control group (Figure 5A). In comparison with IONPs, the *PGC-1α* expression levels were significantly increased in IONPs + Q50 (*p* < 0.05) and IONPs + Q100 (*p* < 0.001). In addition, *PGC-1α* expression levels in IONPs + Q100 were significantly increased (*p* < 0.001) compared with the IONPs + Q25 and IONPs + Q50 groups.

In IONPs (*p* < 0.001), IONPs + Q25 (*p* < 0.001) and IONPs + Q50 (*p* < 0.05), the mRNA expression levels of mitochondrial transcription factor A (*mtTFA*) were significantly decreased than in the control group while they significantly increased in IONPs + Q50 (*p* < 0.01) and IONPs + Q100 (*p* < 0.05) compared with IONPs + Q25. *mtTFA* expression levels were significantly increased (*p* < 0.05) in IONPs + Q100 in comparison with IONPs + Q50 (Figure 5B).

### 2.6. Hematoxylin (H) and Eosin (E) Staining Assessment of Brain Sections

**Figure 6:**
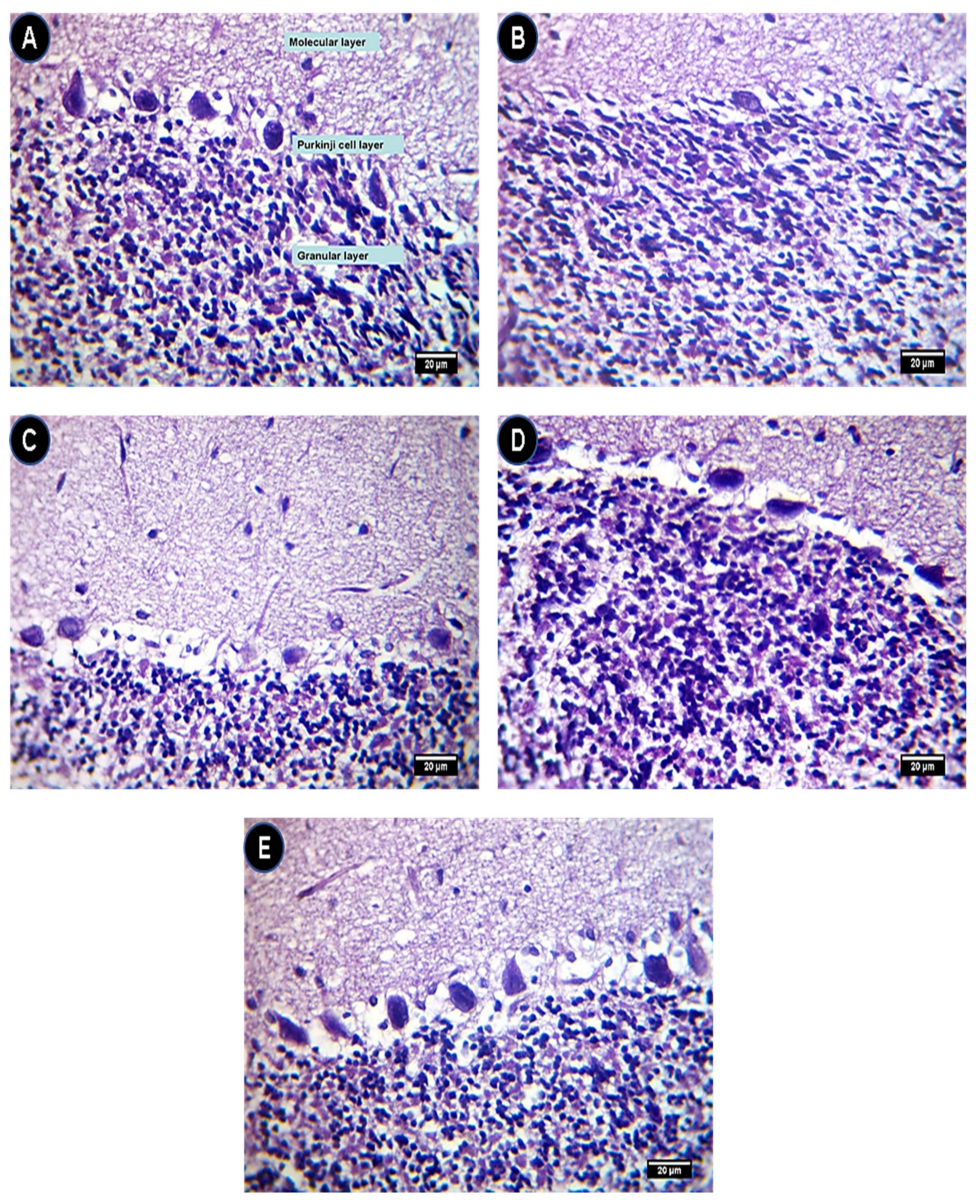
Cerebellum histopathology. (**A**) Control group showing a normal histological structure of cerebellum layers. (**B**) IONP group showing a severe depletion of the Purkinje cells. (**C**) IONPs + Q25 group showing a moderate depletion of the Purkinje cells. (**D**) IONPs + Q50 group showing a moderate to mild depletion of the Purkinje cells. (**E**) IONPs + Q100 group showing a mild depletion of the Purkinje cells. Hematoxylin (H) and eosinH and (E) (×400). Scale bar = 20 µm.

Histological evaluations of brain tissue revealed that the control group showed a normal histological structure of cerebellum layers (Figure 6A). In the IONP group, the cerebellum showed a severe depletion of the Purkinje cell layer (Figure 6B) while this depletion of the Purkinje cell layer due to IONPs was alleviated in the IONPs + Q25 (Figure 6C), IONPs + Q50 (Figure 6D) and IONPs + Q100 (Figure 6E) groups in a dose-dependent manner.

**Scheme 1:**
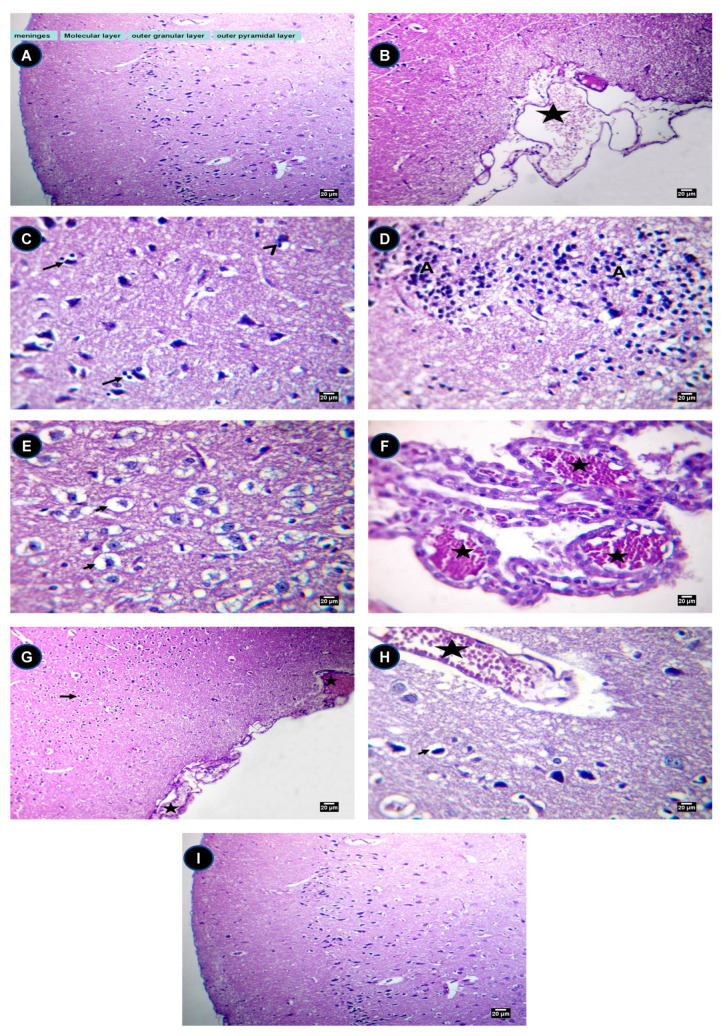
Histopathology assessment. (**A**) The brain of a rat showing the normal histological structure of the meninges and cerebrum cortex, H and E (×200). (**B**) The meninges of a rat treated with IONPs showing the congestion of the submeningeal blood vessels (star), H and E (×200). (**C**) The cerebrum of a rat treated with IONPs showing satellitosis (arrows) and neuronophagia (arrowhead), H and E (×400). (**D**) The cerebrum of a rat treated with IONPs showing gliosis (**A**), H and E (×400). (**E**) The cerebrum of a rat treated with IONPs showing spongiosis (short arrows), H and E (×400). (**F**) The choroid plexus of a rat treated with IONPs showing severe congestion (stars), H and E (×400). (**G**) The brain of a rat in the IONPs + Q25 group showing spongiosis (short arrows) and the congestion of the submeningeal blood vessels (stars), H and E (×200). (**H**) The cerebrum of a rat in the IONPs + Q50 group showing mild spongiosis (short arrows) and the congestion of blood vessels (star), H and E (×400). (**I**) The brain of a rat in the IONPs + Q100 group showing a relatively normal histological structure of the meninges and cerebrum cortex, H and E (×200). Scale bar = 20 µm.

Rats in the control group showed a normal histological structure of the meninges and cerebrum cortex (Scheme 1A) while the meninges of rats treated with IONPs showed congestion of submeningeal blood vessels (Scheme 1B). In the IONP groups, the cerebrum of the brain of the rats showed satellitosis, neuronophagia (Scheme 1C), gliosis (Scheme 1D) and spongiosis (Scheme 1E) besides congestion of the choroid plexus (Scheme 1F).

As can be seen in Scheme 1F, the brain of rats in the IONPs + Q25 group showed spongiosis (short arrows) and congestion of the submeningeal blood vessels. Moreover, the cerebrum of rats in the IONPs + Q50 group showed mild spongiosis and blood vessel congestion (Scheme 1H). The brain of rats in the IONPs + Q100 group showed a relatively normal histological structure of the meninges and cerebrum cortex (Scheme 1I).

### 2.7. Prussian Blue Staining Assessment of Brain Sections

**Figure 7:**
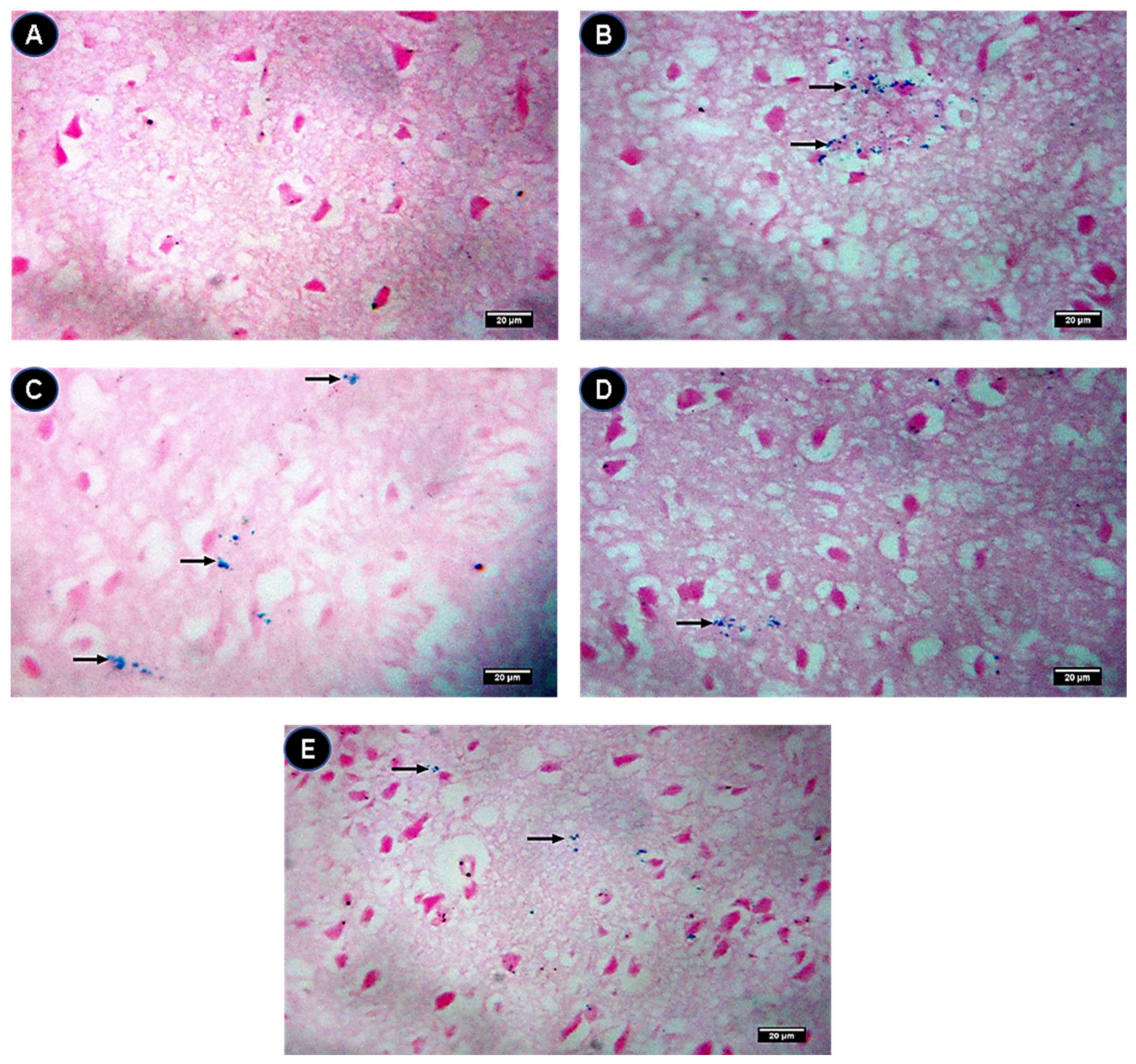
Brain tissue stained with Prussian blue (Black arrows). (**A**) Control group showing a negative Prussian blue stain. (**B**) IONPs group showing blue spots representing the iron contents by Prussian blue stain. (**C**) IONPs + Q25 group showing moderate blue spots representing the iron contents by Prussian blue stain. (**D**) IONPs + Q50 group showing a few blue spots representing the iron contents by Prussian blue stain. (**E**) IONPs + Q100 group showing very few dispersed blue spots representing the iron contents by Prussian blue stain. ×400. Scale bar = 20 µm.

Brain sections of the control group showed negative Prussian blue staining (Figure 7A). In the IONPs group, Prussian blue staining spots were recognized in brain sections (Figure 7B). On the contrary, the intensity of the Prussian blue staining spots of the sections of the IONPs group was attenuated by quercetin in the IONPs + Q25 (Figure 7C), IONPs + Q50 (Figure 7D) and IONPs + Q100 (Figure 7E) groups in a dose-dependent manner.

### 2.8. Caspase 3 and Bcl2 Protein Levels in Brain Sections

**Figure 8:**
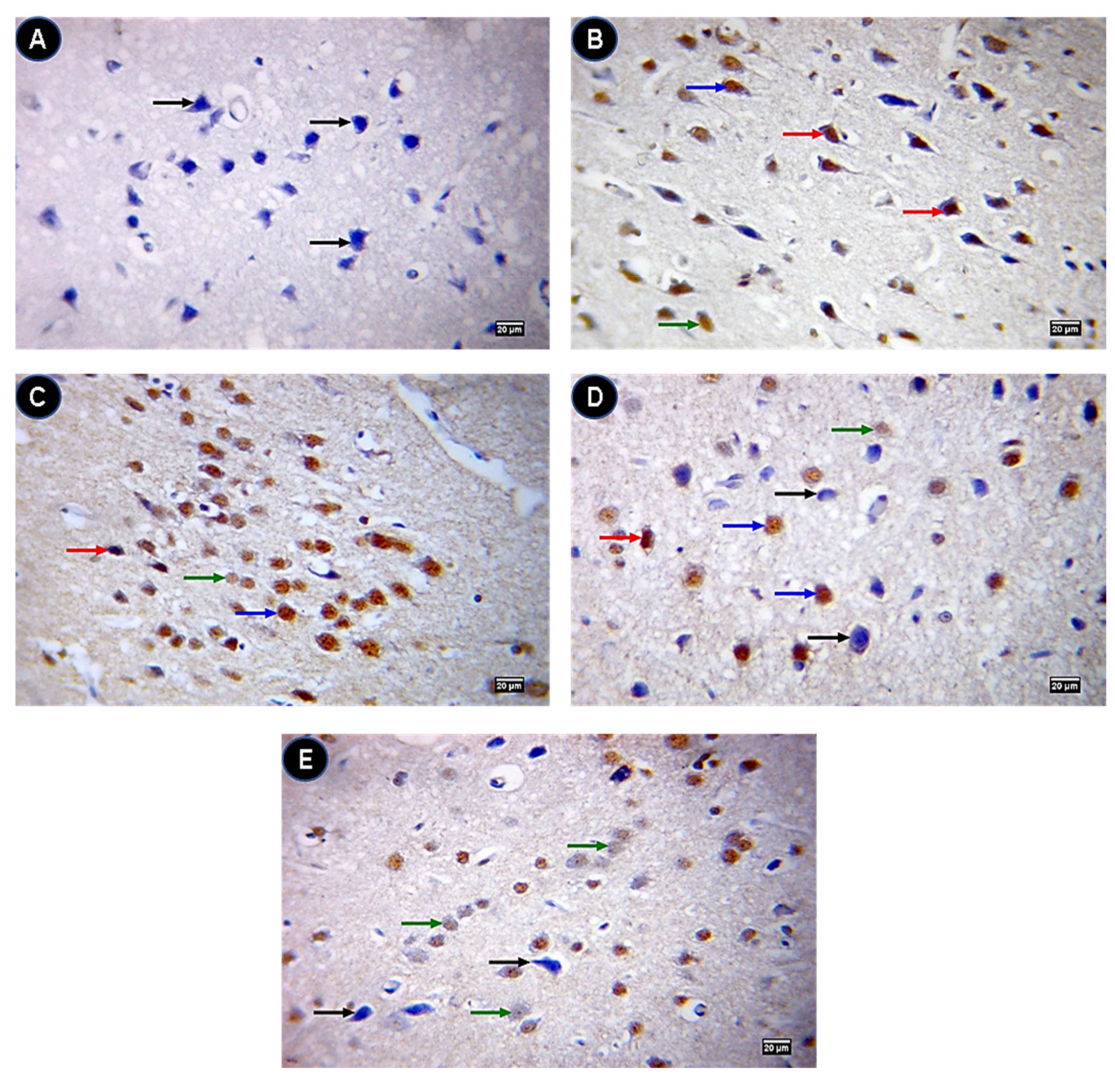
Immunohistochemistry of caspase 3 in brain tissue. (**A**) Control group showing a caspase 3 negative (black arrow) immunostained neuron. (**B**) IONPs group showing strong (red arrows), moderate (blue arrow) and weak (green arrow) caspase 3 positive brown immunostained neurons. (**C**) IONPs + Q25 group showing strong (red arrow), moderate (blue arrow) and weak (green arrow) caspase 3 positive brown immunostained neurons. (**D**) IONPs + Q50 group showing strong (red arrow), moderate (blue arrows) and weak (green arrow) caspase 3 positive brown immunostained neurons. (**E**) IONPs + Q100 group showing negative (black arrows) and weak (green arrow) caspase 3 positive brown immunostained neurons. ×400. Scale bar = 20 µm.

**Figure 9:**
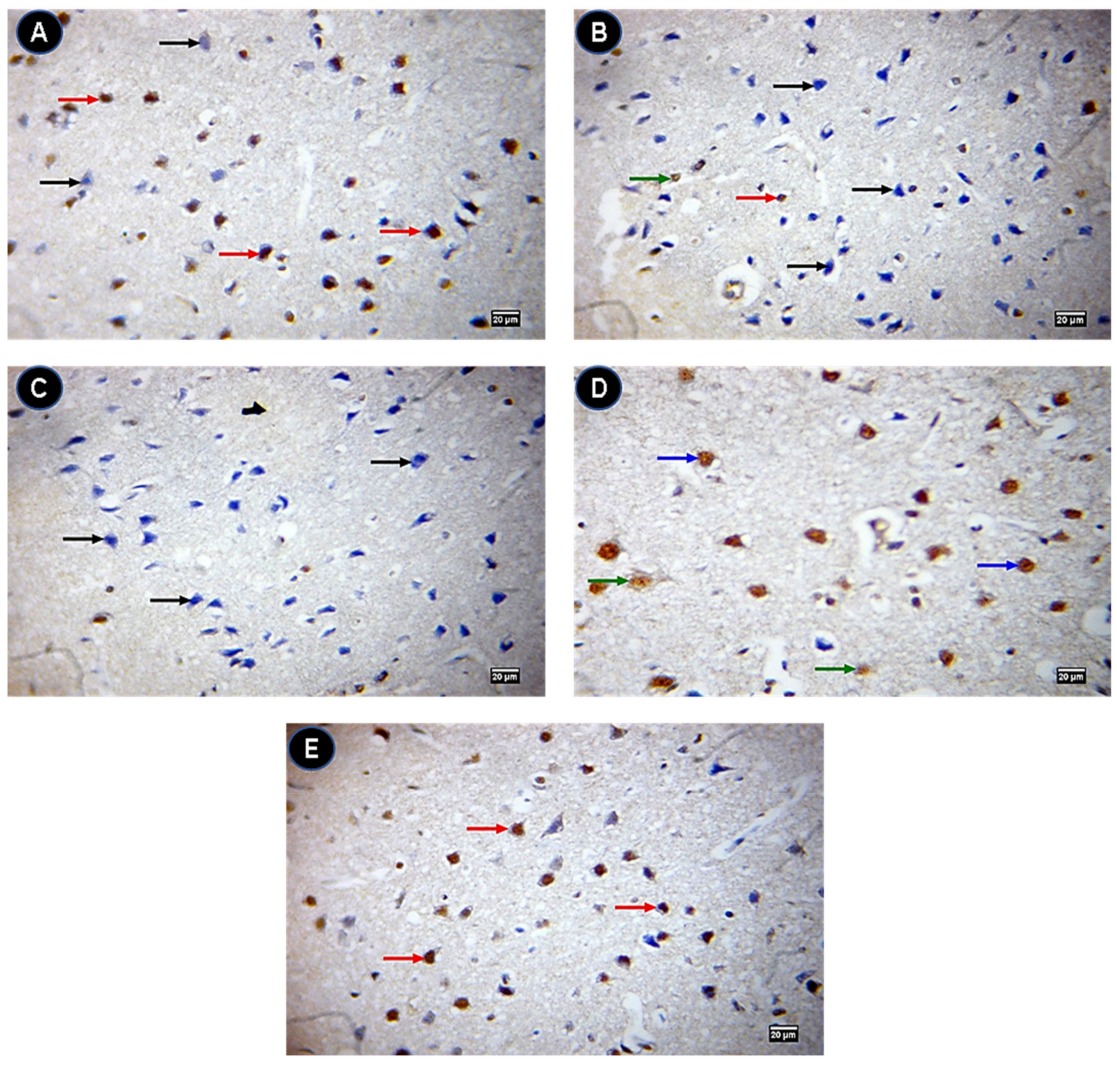
Immunohistochemistry of Bcl2 in brain tissue. (**A**) Control group showing negative (black arrow) and strong (red arrow) Bcl2 positive brown immunostained neurons. (**B**) IONPs group showing negative (black arrows) and a few strong (red arrows) and weak (green arrow) Bcl2 positive brown immunostained neurons. (**C**) IONPs + Q25 group showing negative (black arrows) Bcl2 brown immunostained neurons. (**D**) IONPs + Q50 group showing moderate (blue arrows) and weak (green arrows) Bcl2 positive brown immunostained neurons. (**E**) IONPs + Q100 group showing strong (red arrows) Bcl2 positive brown immunostained neurons. ×400. Scale bar = 20 µm.

The control group showed a negative expression of caspase 3 protein levels (Figure 8A) while they were highly expressed in IONPs (Figure 8B). Quercetin-treated groups (Figure 8C–E) showed a low expression of caspase 3 compared with IONPs. On the contrary, Bcl2 was significantly expressed in IONPs + Q25 (Figure 9C), IONPs + Q50 (Figure 9D) and IONPs + Q100 (Figure 9E) compared with IONPs (Figure 9B) and the control group (Figure 9A).

## 3. Discussion

Exposure to IONPs led to iron and its deposition in soft tissues especially in the brain [21]. In the present study, we recognized iron deposition in the brain evidenced by Prussian blue staining and morphology alterations monitored by the H and E staining assessment. Dhakshinamoorthy et al. [22] demonstrated that iron content was increased significantly in brain tissue of IONP-treated groups compared with the control. This was assessed by Prussian blue staining of the brain regions and evidenced by blue spots in the frontal cortex, hippocampus and cerebellum. El-Sayed et al. [23] also stated significant rises in iron levels in brain tissue due to IONP administration in rats.

IONPs could have more capability to penetrate the BBB and induce brain cell injuries more than other organ cells [24,25]. Another explanation may be that increased iron levels in the brain due to the binding of iron to transferrin triggers the upregulation of iron receptors in the brain and, consequently, transports iron across the BBB [26].

Free Fe^3+^ ions react with H_2_O_2_ to generate a ROS in the Fenton reaction process [27]. The elevated ROS increased the permeability of the outer mitochondrial membrane, the lipid peroxidation, the protein damage and the DNA chain broke [28]. The oxidative stress of the ROS led to increases in the brain levels of the lipid peroxidation product, MDA, as stated in the current study in IONP-treated rats. Similarly, Dhakshinamoorthy et al. [22] reported significant increases in MDA levels in brain tissues of IONP-treated mice. Reddy et al. [26] also stated that the MDA levels were significantly increased in brain tissue in a high dose but a non-significant increase in a low dose in IONP-treated rats. Gaharwar and Paulraj [5] revealed that MDA levels were significantly elevated in IONP-treated rats. Similarly, IONPs induced the oxidative injury of cardiomyocytes monitored by a high MDA production and diminished GSH concentrations [29].

Authors in several studies have investigated the protective effect of natural products or their extracts against the side effects associated with IONPs including *Echinacea purpurea* [30] and *Anthriscus sylvestris* [31] extracts. In the current study, we investigated the protective role of quercetin against the toxicity of IONPs in the brains of rats. Quercetin supplementation to IONP-treated rats decreased the MDA levels in brain tissue and our results were in concordance with those obtained by Dong et al. [32] who stated that quercetin decreased lipid peroxidation by lowering the MDA levels in brain tissue in rats. This may be explained by the ability of quercetin to reduce the ROS therefore inhibiting lipid peroxidation and preventing the formation of MDA [33]. This was attributed to the catechol group (B ring) and the OH group at position 3 of the A and C ring, which have optimal free-radical scavenging [17].

GSH is an essential part of the cellular anti-oxidant defense that directly reacts with the ROS and other reactive species [34]. In the present study, brain GSH levels were significantly decreased in IONP-treated rats but GSSG levels were significantly increased. Reddy et al. [26] stated the depletion of GSH levels in brain tissue of IONP-treated rats and suggested that it might be due to an increased utilization of GSH in conjugation reactions as part of a detoxification mechanism, therefore reduced GSH decreased and oxidized GSSG increased. The co-administration of IONP-treated rats with quercetin restored that by increasing GSH and decreasing GSSG levels in brain tissue. These results were in harmony with Singh et al. [35] who reported the elevation of GSH levels in brain tissue indicating quercetin’s anti-oxidant potential. Dong et al. [32] also revealed that quercetin altered the nuclear factor erythroid 2-related factor 2 (Nrf2) gene expression. Nrf2 consequently stimulated the anti-oxidant enzyme production in brain tissues.

In the present study, CPK was significantly increased in brain tissue of IONP-treated rats. In cellular energy buffering and energy transmission, CPK/phosphocreatine plays a major role especially in cells with high and fluctuating energy needs such as neurons [36]. Therefore, activation of the CPK/phosphocreatine system and CPK expression changes may be an earlier indicator of oxidative and bioenergetic stress in the cell [37] as compensation for a decreasing energy production due to oxidative stress. The co-administration of IONP-treated rats with quercetin decreased CPK in brain tissue. Lemmens et al. [38] demonstrated decreases of CPK activities due to quercetin supplementation.

AChE activity in nervous tissue is responsible for the hydrolysis of acetylcholine (Ach) to choline at the synapses and the neuromuscular junction [39]. In the present study, AChE was significantly increased in IONP-treated rats and this result was in harmony with Dhakshinamoorthy et al. [22] who recognized significant increases in AChE activities in brain tissue of IONP-treated rats in which iron accumulation by IONPs altered the cholinergic system.

Neurotransmitters are endogenous chemical substances that allow neurons to communicate throughout the body; they enable the brain to perform different functions in the chemical synaptic transmission [40]. In the current study, epinephrine, serotonin and melatonin concentrations in brain tissues were significantly decreased in IONP-treated rats. Yousef et al. [41] reported that serotonin and dopamine levels were significantly decreased in brain tissue in IONP-treated rats. In contrast, quercetin increased epinephrine, serotonin and melatonin concentrations in brain tissues compared with IONPs. Singh et al. [35] stated that quercetin increased serotonin, norepinephrine and dopamine.

Mitochondrial biogenesis assumes a principal role in maintaining mitochondrial homeostasis to meet the cellular physiological needs of neural cells [42]. *PGC-1α* is a co-transcriptional regulation factor that induces mitochondrial biogenesis and promotes the expression of *mtTFA* [43]. In the present study, the expression levels of *PGC-1α* and *mtTFA* were significantly decreased in brain tissue in IONP-treated rats. Our results were in concordance with those obtained by Yousef et al. [44]. They illustrated that mitochondrial biogenesis represented by *PGC-1α* and *mtTFA* significantly decreased their expression in brain tissue in IONP-exposed rats indicating a decreased mitochondrial biogenesis and *mtDNA* replication and transcription that could lead to mitochondrial dysfunction. The co-administration of IONP-treated rats with quercetin restored this decrease by elevating both *PGC-1α* and *mtTFA* expressions in brain tissue and our results were in harmony with Sharma et al. [45] who reported that quercetin increased the expression of *PGC-1α*, *NRF-1*, *NRF-2* and *mtTFA*. They suggested that due to a decreasing ROS by the quercetin induced *PGC-1α* raising, this led to the activation of *NRF-1* and *NRF-2* stimulated expression of *mtTFA*.

Quercetin attenuated the apoptotic effect of IONPs through a significant reduction in caspase 3 and increase in Bcl2 expression levels in brain immunohistochemical sections. This finding supports the anti-oxidant potential of quercetin. Similarly, quercetin treatment attenuated the increase of caspase 3 in ischemic brain injury in rats [46,47] and Bcl2 in lipopolysaccharide-treated mice [48].

## 4. Materials and Methods

### 4.1. Reagents and Chemicals

Iron (III) oxide nanoparticle powder was obtained from Sigma–Aldrich (Louis, Mo, USA), which was solubilized in deionized water before use. Quercetin in a powdered form was also obtained from Sigma–Aldrich, which was dissolved in dimethyl sulfoxide (DMSO) and distilled water at a ratio of 1:20, respectively, before use. DMSO ≥ 99.6% was purchased from Sigma–Aldrich. Other chemicals and deionized water were bought from the Center for Graduate Studies and Research at Alexandria University.

### 4.2. Ethics Statement

The study was approved in response to the guidelines of “NIH Guide for the Care and Use of Laboratory Animals” by the Faculty of Veterinary Medicine Ethics Committee of Alexandria University, Egypt.

### 4.3. Characterization of IONPs

The morphology and the particle size of IONPs were examined by scanning electron microscope (SEM). The particle’s charge was also determined by zeta potential using a ZetaSizer Nano ZS (Malvern Instruments, Malvern, UK).

### 4.4. Animals, Housing and Experimental Design

Forty healthy adult male albino rats weighing 150 ± 20 body weight (B.W.) were purchased from the Animal Breeding Unit, Medical Research Institute of Alexandria University, Egypt. The animals were kept in metal cages under controlled environmental conditions with an optimum temperature (23 ± 2), humidity (55 ± 5) and a dark/light cycle (12 h) and free access to a basal feed diet (Table 1) and drinking water. All animals were housed for two weeks before the experiment for acclimatization. The rats were randomly assigned to five groups (eight rats each); the control received a normal basal diet and water ad libitum, the IONPs group was intraperitoneally injected with IONPs 50 mg/kg B.W. three times a week [49], the IONPs + Q25 mg group was administrated with the same dose of IONPs and gavaged with 25 mg quercetin/kg B.W. daily, the IONPs + Q50 group was administrated with the same dose of IONPs and gavaged with 50 mg quercetin/kg B.W. daily and the IONPs + Q100 group was administrated with the same dose of IONPs and gavaged with 100 mg quercetin/kg B.W. daily [17]. All treatments were maintained for 30 days.

### 4.5. Sampling

At the end of the experiment, the rats were fasted for 12 h and anesthetized using an intraperitoneal injection of ketamine/xylazine (100 mg/kg/10 mg/kg) then euthanized and the brain was immediately dissected, rinsed with chilled normal saline 0.9% and divided into three parts; the first one was used for biochemical analyses. The second part was kept in −80 °C for mRNA extraction and RT-PCR assessment while the last part was flushed with phosphate buffer saline (PBS, pH 7.4) and fixed in 4% paraformaldehyde dissolved in PBS for 48 h for the sample fixation.

### 4.6. Biochemical Analyses

Parts of each rat brain were homogenized in cold phosphate buffer saline (PBS) and centrifuged for 10 min at 4 °C at 1435 × *g*. Malondialdehyde (MDA), GSH, oxidized glutathione (GSSG) and creatine phosphokinase (CPK) were determined by the commercial kits of Biodiagnostic Co. (Giza, Egypt). Acetylcholinesterase (AChE) enzyme activities in brain homogenates were determined by a colorimetric assay kit (BioVision Co., Milpitas, CA, USA). Epinephrine, serotonin and melatonin levels in brain homogenates were also determined using ELISA kits (BioVision Co., Milpitas, CA, USA).

### 4.7. mRNA Extraction and RT-PCR

Total RNA was extracted from the samples using the manufacturer’s easy-RED Total RNA Extraction Kits (iNtRON Biotechnology, Inc., Gyeonggi-do, South Korea). The first-strand cDNA was achieved using the HiSen Script cDNA (iNtRON Biotechnology, Inc.) package. Specific primers were used to amplify selected genes with glyceraldehyde 3-phosphate dehydrogenase (*GAPDH*) as a stable housekeeping gene (Table 2). mRNA expression was achieved using a real-time Strata gene MX3005P PCR (Agilent Technologies, Santa Clara, CA, USA) and a TOP real TM PreMIX SYBR Green qPCR master blend (cat. RT 500, Enzynomics, Daejeon, South Korea) following the manufacturer’s instructions. The relative gene expression concentrations were evaluated using the 2^−ΔΔct^ method as described by Pfaffl [50].

### 4.8. Histopathological Examination

Using the traditional paraffin incorporation technique, the fixed specimens were dehydrated by ascending degrees of ethanol, cleared into three xylene shifts and were finished by paraffin embedding at 65 °C. Sections 4 µm thick were stained with Hematoxylin and Eosin (H and E) [51].

Brain sections were hydrated through a series of decreasing alcohol concentrations and placed in a 20% potassium ferrocyanide solution with a 20% HCl solution for 30 min. The slides were washed, dehydrated, cleaned with xylene and observed under the microscope for blue staining of iron contents [52].

### 4.9. Immunohistochemical Examination

The standard horseradish peroxidase (HRP) immunohistochemistry technique was applied to the positively charged slides of the paraffin tissue sections. According to the manufacturer’s guidelines, rabbit anti-rat caspase 3 (Lab Vision, Fremont, CA, USA) and Bcl2 (DAKO, Glostrup, Denmark) were used. Five µm-thick sections of the cerebrum, cerebellum, spinal cord and sciatic nerve were dewaxed, rehydrated and pretreated with 3% hydrogen peroxide (H_2_O_2_) to block endogenous peroxidase activity. Antigen retrieval was accomplished by placing slides in a microwave for 10 min in a 10 mM sodium citrate buffer (pH 6.0). Slides were incubated with the primary antibody and then rinsed with Tris-buffer saline and the secondary antibody. Slides were incubated with a 3, 3′-diaminobenzidine (DAB) substrate chromogen solution and then counterstained with Mayer’s hematoxylin. Images of 10 different fields were imaged at a magnification of ×400.

### 4.10. Statistical Analysis

A one-way ANOVA with Tukey’s post hoc multiple range test were used for the data analysis using a GraphPad Prism v.5 https://www.graphpad.com/, accessed on 10 March 2021 (GraphPad, San Diego, CA, USA). All declarations of significance depended on *p* < 0.05.

## 5. Conclusions

Iron oxide nanoparticles (IONPs) intraperitoneally injected by a dose of 50 mg/kg B.W. induced oxidative alterations in the brain while quercetin in doses of 25, 50 and 100 mg/kg B.W. alleviated brain oxidative injuries induced by IONPs. Therefore, quercetin is a promising feed supplement along with IONP therapy.

## Figures and Tables

**Table 1:** Ingredients of the basal diet.

g/kg Diet	Ingredients
529.5	Corn flour
200	Casein
100	Sucrose
70	Soybean oil
50	Cellulose
35	Mineral mix
10	Vitamin mix
3	l-cystine
2.5	Choline

**Table 2:** Sequences of primers.

Gene	Primer Sequence (5′→3′)	
***PGC-1α***	**F:**	AAACTTGCTAGCGGTCCTCA
	**R:**	TGGCTGGTGCCAGTAAGAG
***mTFA***	**F:**	CCCTGGAAGCTTTCAGATACG
	**R:**	AATTGCAGCCATGTGGAGG
***GAPDH***	**F:**	GGGTGTGAACCACGAGAAATA
	**R:**	AGTTGTCATGGATGACCTTGG

*GAPDH*; glyceraldehyde 3-phosphate dehydrogenase, *PGC-1α*; peroxisome proliferator-activated receptor gamma coactivator 1-alpha, *mTFA*; mitochondrial transcription factor A.

## Data Availability

Data is contained within the article.

## References

[1] Mazdeh M., Rahiminejad M.E., Nili-Ahmadabadi A., Ranjbar A. (2016). Neurological Disorders and Oxidative Toxic Stress: A Role of Metal Nanoparticles. Jundishapur J. Nat. Pharm. Prod..

[2] Fidler M.C., Walczyk T., Davidsson L., Zeder C., Sakaguchi N., Juneja L.R., Hurrell R.F. (2004). A micronised, dispersible ferric pyrophosphate with high relative bioavailability in man. Br. J. Nutr..

[3] Ramimoghadam D., Bagheri S., Hamid S.B.A. (2014). Progress in electrochemical synthesis of magnetic iron oxide nanoparticles. J. Magn. Magn. Mater..

[4] Gaharwar U.S., Kumar S., Rajamani P. (2020). Iron oxide nanoparticle-induced hematopoietic and immunological response in rats. RSC Adv..

[5] Gaharwar U.S., Paulraj R. (2015). Iron Oxide Nanoparticles Induced Oxidative Damage in Peripheral Blood Cells of Rat. J. Biomed. Sci. Eng..

[6] Feng Q., Liu Y., Huang J., Chen K., Huang J., Xiao K. (2018). Uptake, distribution, clearance, and toxicity of iron oxide nanoparticles with different sizes and coatings. Sci. Rep..

[7] Gaharwar U.S., Meena R., Rajamani P. (2019). Biodistribution, clearance and morphological alterations of intravenously administered iron oxide nanoparticles in male wistar rats. Int. J. Nanomed..

[8] Palmieri B., Sblendorio V. (2007). Oxidative stress tests: Overview on reliability and use, Part II. Eur. Rev. Med. Pharmacol. Sci..

[9] Naqvi S., Samim M., Abdin M.Z., Ahmed F.J., Maitra A.N., Prashant C.K., Dinda A.K. (2010). Concentration-dependent toxicity of iron oxide nanoparticles mediated by increased oxidative stress. Int. J. Nanomed..

[10] David A.V.A., Arulmoli R., Parasuraman S. (2016). Overviews of biological importance of quercetin: A bioactive flavonoid. Pharmacogn. Rev..

[11] Wu W., Li R., Li X., He J., Jiang S., Liu S., Yang J. (2015). Quercetin as an Antiviral Agent Inhibits Influenza A Virus (IAV) Entry. Viruses.

[12] Akdemir F.N.E., Gülçin İ., Karagöz B., Soslu R. (2016). Quercetin protects rat skeletal muscle from ischemia reperfusion injury. J. Enzym. Inhib. Med. Chem..

[13] Barcelos G.R.M., Angeli J.P.F., Serpeloni J.M., Grotto D., Rocha B.A., Bastos J.K., Knasmüller S., Júnior F.B. (2011). Quercetin protects human-derived liver cells against mercury-induced DNA-damage and alterations of the redox status. Mutat. Res. Genet. Toxicol. Environ. Mutagen..

[14] Salem I.B., Boussabbeh M., Graiet I., Rhouma A., Bacha H., Essefi S.A. (2016). Quercetin protects HCT116 cells from Dichlorvos-induced oxidative stress and apoptosis. Cell Stress Chaperones.

[15] Xu X.R., Yu H.T., Yang Y., Hang L., Yang X.W., Ding S.H. (2016). Quercetin phospholipid complex significantly protects against oxidative injury in ARPE-19 cells associated with activation of Nrf2 pathway. Eur. J. Pharmacol..

[16] Periasamy R., Kalal I.G., Krishnaswamy R., Viswanadha V. (2016). Quercetin protects human peripheral blood mononuclear cells from OTA-induced oxidative stress, genotoxicity, and inflammation. Environ. Toxicol..

[17] El-Far A.H., Lebda M.A., Noreldin A.E., Atta M.S., Elewa Y.H.A., Elfeky M., Mousa S.A. (2020). Quercetin Attenuates Pancreatic and Renal D-Galactose-Induced Aging-Related Oxidative Alterations in Rats. Int. J. Mol. Sci..

[18] Ferri P., Angelino D., Gennari L., Benedetti S., Ambrogini P., Del Grande P., Ninfali P. (2015). Enhancement of flavonoid ability to cross the blood-brain barrier of rats by co-administration with α-tocopherol. Food Funct..

[19] Lesjak M., Srai S.K.S. (2019). Role of dietary flavonoids in iron homeostasis. Pharmaceuticals.

[20] Bagad M., Khan Z.A. (2015). Poly(n-butylcyanoacrylate) nanoparticles for oral delivery of quercetin: Preparation, characterization, and pharmacokinetics and biodistribution studies in Wistar rats. Int. J. Nanomed..

[21] Luo S., Ma C., Zhu M.Q., Ju W.N., Yang Y., Wang X. (2020). Application of Iron Oxide Nanoparticles in the Diagnosis and Treatment of Neurodegenerative Diseases with Emphasis on Alzheimer’s Disease. Front. Cell. Neurosci..

[22] Dhakshinamoorthy V., Manickam V., Perumal E. (2017). Neurobehavioural Toxicity of Iron Oxide Nanoparticles in Mice. Neurotox. Res..

[23] El-Sayed E.H.K., Mohammed Z.A., Ahmed M.M. (2019). Ameliorative role of quercetin in iron overload induced heart and brain toxicity in adult male albino rats. J. Toxicol. Environ. Health Sci. Full Length Res. Pap..

[24] Laurent S., Burtea C., Thirifays C., Häfeli U.O., Mahmoudi M. (2012). Crucial Ignored Parameters on Nanotoxicology: The Importance of Toxicity Assay Modifications and “Cell Vision”. PLoS ONE.

[25] Mahmoudi M., Laurent S., Shokrgozar M.A., Hosseinkhani M. (2011). Toxicity evaluations of superparamagnetic iron oxide nanoparticles: Cell “vision” versus physicochemical properties of nanoparticles. ACS Nano.

[26] Reddy U.A., Prabhakar P.V., Mahboob M. (2017). Biomarkers of oxidative stress for in vivo assessment of toxicological effects of iron oxide nanoparticles. Saudi J. Biol. Sci..

[27] Wu H., Yin J.J., Wamer W.G., Zeng M., Lo Y.M. (2014). Reactive oxygen species-related activities of nano-iron metal and nano-iron oxides. J. Food Drug Anal..

[28] Sharma P., Jha A.B., Dubey R.S., Pessarakli M. (2012). Reactive Oxygen Species, Oxidative Damage, and Antioxidative Defense Mechanism in Plants under Stressful Conditions. J. Bot..

[29] Shen Y., Gong S., Li J., Wang Y., Zhang X., Zheng H., Zhang Q., You J., Huang Z., Chen Y. (2019). Co-loading antioxidant N-acetylcysteine attenuates cytotoxicity of iron oxide nanoparticles in hypoxia/ reoxygenation cardiomyocytes. Int. J. Nanomed..

[30] Awaad A., Adly M.A., Hosny D. (2018). Insulin-like 3 expression and fibrosis induction after intra-testicular injection of magnetic nanoparticles in rat testis and the ameliorative role of *Echinacea purpurea* extract. Biotech. Histochem..

[31] Negut I., Grumezescu V., Grumezescu A.M., Bîrcă A.C., Holban A.M., Urzica I., Avramescu S.M., Gălățeanu B., Hudiță A. (2020). Nanostructured Thin Coatings Containing *Anthriscus sylvestris* Extract with Dual Bioactivity. Molecules.

[32] Dong Y.S., Wang J.L., Feng D.Y., Qin H.Z., Wen H., Yin Z.M., Gao G.D., Li C. (2014). Protective effect of quercetin against oxidative stress and brain edema in an experimental rat model of subarachnoid hemorrhage. Int. J. Med. Sci..

[33] Uzun F.G., Kalender Y. (2013). Chlorpyrifos induced hepatotoxic and hematologic changes in rats: The role of quercetin and catechin. Food Chem. Toxicol..

[34] Siems W., Crifo C., Capuozzo E., Uchida K., Grune T., Salerno C. (2010). Metabolism of 4-hydroxy-2-nonenal in human polymorphonuclear leukocytes. Arch. Biochem. Biophys..

[35] Singh S., Jamwal S., Kumar P. (2017). Neuroprotective potential of Quercetin in combination with piperine against 1-methyl-4-phenyl-1,2,3,6-tetrahydropyridine-induced neurotoxicity. Neural Regen. Res..

[36] Andres R.H., Ducray A.D., Schlattner U., Wallimann T., Widmer H.R. (2008). Functions and effects of creatine in the central nervous system. Brain Res. Bull..

[37] Venkataraman P., Krishnamoorthy G., Selvakumar K., Arunakaran J. (2009). Oxidative stress alters creatine kinase system in serum and brain regions of polychlorinated biphenyl (Aroclor 1254)-exposed rats: Protective role of melatonin. Basic Clin. Pharmacol. Toxicol..

[38] Lemmens K., Vrolijk M., Bouwman F., van der Vijgh W., Bast A., Haenen G. (2014). The Minor Structural Difference between the Antioxidants Quercetin and 4′O-Methylquercetin Has a Major Impact on Their Selective Thiol Toxicity. Int. J. Mol. Sci..

[39] Kumari M., Rajak S., Singh S.P., Kumari S.I., Kumar P.U., Murty U.S.N., Mahboob M., Grover P., Rahman M.F. (2012). Repeated oral dose toxicity of iron oxide nanoparticles: Biochemical and histopathological alterations in different tissues of rats. J. Nanosci. Nanotechnol..

[40] Rizo J. (2018). Mechanism of neurotransmitter release coming into focus. Protein Sci..

[41] Yousef M.I., Abuzreda A.A., Kamel M.A.E.-N. (2019). Neurotoxicity and inflammation induced by individual and combined exposure to iron oxide nanoparticles and silver nanoparticles. J. Taibah Univ. Sci..

[42] Zhao Q., Tian Z., Zhou G., Niu Q., Chen J., Li P., Dong L., Xia T., Zhang S., Wang A. (2020). SIRT1-dependent mitochondrial biogenesis supports therapeutic effects of resveratrol against neurodevelopment damage by fluoride. Theranostics.

[43] Puigserver P., Wu Z., Park C.W., Graves R., Wright M., Spiegelman B.M. (1998). A cold-inducible coactivator of nuclear receptors linked to adaptive thermogenesis. Cell.

[44] Yousef M., Abuzreda A., Kamel M. (2019). Cardiotoxicity and lung toxicity in male rats induced by long-term exposure to iron oxide and silver nanoparticles. Exp. Ther. Med..

[45] Sharma D.R., Sunkaria A., Wani W.Y., Sharma R.K., Verma D., Priyanka K., Bal A., Gill K.D. (2015). Quercetin protects against aluminium induced oxidative stress and promotes mitochondrial biogenesis via activation of the PGC-1α signaling pathway. Neurotoxicology.

[46] Park D.J., Jeon S.J., Kang J.B., Koh P.O. (2020). Quercetin Reduces Ischemic Brain Injury by Preventing Ischemia-induced Decreases in the Neuronal Calcium Sensor Protein Hippocalcin. Neuroscience.

[47] Pei B., Yang M., Qi X., Shen X., Chen X., Zhang F. (2016). Quercetin ameliorates ischemia/reperfusion-induced cognitive deficits by inhibiting ASK1/JNK3/caspase-3 by enhancing the Akt signaling pathway. Biochem. Biophys. Res. Commun..

[48] Khan A., Ali T., Rehman S.U., Khan M.S., Alam S.I., Ikram M., Muhammad T., Saeed K., Badshah H., Kim M.O. (2018). Neuroprotective effect of quercetin against the detrimental effects of LPS in the adult mouse brain. Front. Pharmacol..

[49] Sundarraj K., Manickam V., Raghunath A., Periyasamy M., Viswanathan M.P., Perumal E. (2017). Repeated exposure to iron oxide nanoparticles causes testicular toxicity in mice. Environ. Toxicol..

[50] Pfaffl M.W. (2001). A new mathematical model for relative quantification in real-time RT-PCR. Nucleic Acids Res..

[51] Bancroft J.D., Layton C., Suvarna S.K., Layton C., Bancroft J.D. (2013). The hematoxylin and eosin. Theory Practice of Histological Techniques.

[52] Glat M., Skaat H., Menkes-Caspi N., Margel S., Stern E.A. (2013). Age-dependent effects of microglial inhibition in vivo on Alzheimer’s disease neuropathology using bioactive-conjugated iron oxide nanoparticles. J. Nanobiotechnol..

